# Some Remarks on the Therapeutics of Ferratin and Lactophenin

**Published:** 1896-12

**Authors:** T. V. Hubbard

**Affiliations:** Atlanta, Ga., Assistant to Chair of Materia Medica and Therapeutics, Atlanta Medical College


### SOME REMARKS ON THE THERAPEUTICS OF FERRATIN AND LACTOPHENIN.

BY T. V. HUBBARD, M.D., ATLANTA, GA.,

Assistant to Chair of Materia Medica and Therapeutics, Atlanta Medical College.

The profession is well aware of the fact that the administration of the inorganic salts of iron does not always give satisfactory results, and the pharmacists have been for some time endeavoring to secure an iron compound which would not only meet the indications required and relieve the condition for which it was administered, but do so quickly and pleasantly, without the production of the undesirable symptoms of headache, constipation, and disturbance of the stomach which so often accompanies the administration of these agents. So far as my experience and observation go I

think we have in the preparation known as ferratin a therapeutic agent which is destined to supplant to a large extent the inorganic salts of iron. It is a rusty colored powder, insoluble in water, but soluble in alcoholic solutions, and can be administered in tablet or powder form, as it is practically odorless and tasteless. The dose is from seven and one-half to fifteen grains three times daily. Ferratin is extracted from the liver of the pig and is supposed to represent the natural form in which iron is taken into the system with food. It is easily absorbed, does not produce headache, constipation, or gastric irritation, in striking contrast to other preparations of iron. The therapeutic importance of ferratin is based on the fact that it is absorbed and stored up in the liver where it is available for immediate use, while other preparations, after a slow and difficult absorption, must undergo a change into ferratin before they are active agents for the supply of the organism with the amount of natural iron necessary to its growth and nutrition. Germain Sée has administered ferratin in a number of cases and finds that it increases the nutrition of the tissues without causing any gastric disturbances, even after prolonged administration. He observed that it acted as a mild astringent without constipating the bowels, and increased the appetite. He found it indicated in anemia, chlorosis, and in all conditions of malnutrition where there was a diminution of red blood corpuscles. Dr. Samuel Wolfe, of Philadelphia, reports a case in the *New York Medical Journal* of a woman thirty-seven years old who was suffering from aneurismal dilatation of right carotid, who after an abortion and speedy convalescence, again became pregnant and went to full term without any specially untoward symptoms. In the second week after delivery she began to show decided signs of anemia. She was given the ordinary preparations of iron for several weeks, but gained very little, and the breast milk was lessening so much that she was on the point of substituting artificial food. After the use of ferratin, one gramme daily, the improvement was decided. In five days the anemia disappeared and the milk returned. The patient recovered after the use of ferratin four weeks.

The writer's experience with ferratin is rather favorable and perfectly coincides with the above reports. It was tried in doses of seven and one-half grains three times a day, on a chlorotic, anemic girl, who suffered with headache and dysmenorrhea, and showed decided anemia. After two weeks' administration her appetite improved, her headache has disappeared, and the color has returned to her lips and cheeks, showing marked improvement in the number and quality of the red blood corpuscles. She has also gained in weight.

Another case of a young man suffering from chronic malarial poisoning with its concomitant symptoms, pallor and anemic skin, pains in the back and extremities, headache, dizziness on exertion, was given ferratin in above doses with very decided improvement in all of his disagreeable symptoms after two weeks' adminstration of this agent. Other cases might be reported and other authorities referred to, but I think the above sufficient to demonstrate to any unprejudiced mind the availability of ferratin as a remedial measure, and thus secure for it a conspicuous place in the therapeutics of to-day. It has been tested by authorities of national reputation, and they have given it their unqualified indorsement as a scientific agent capable of increasing the red blood corpuscles and hemoglobin, and improving appetite and body weight.

*Lactophenin.*—As there is such a prolific production at the present day of antipyretics and analgesics of the coal-tar variety the physician is often at a loss to know which one will best meet the indications required in each particular case. It is in behalf of a drug which I consider worthy their consideration and trial that I offer these few remarks. Lactophenin is a white crystalline powder soluble in water and practically tasteless, doses four to eight grains, may be administered in capsules or powder. I have found that lactophenin, in above doses, reduces abnormally high temperature without any of the undesirable symptoms which follow the administration of antipyrine and acetanilid, namely, marked depression of the circulation and respiration with cyanosis. Lactophenin not only reduces temperature, but it relieves more promptly and efficiently the pain, and other nervous symptoms, which accompany fever, to a greater extent than I have been able to secure by the administration of any of the other drugs of this class. While I do not consider it superior to phenacetine as a pure antipyretic, yet for the relief of those symptoms which so often accompany a fever of any kind, such as headache and delirium, pains in the back and extremities, I think lactophenin deserves first place. I have tried it in congestive headache and found that it gave prompt relief and produced a mild hypnosis. I think it a drug that deserves at least a fair trial at the hands of the profession.

*Fitten Building.*

---

### SURGICAL HINTS.

In the *International Journal of Surgery* the following excellent hints are given: When a wound, either accidental or operative, shows signs of infection, never wait for suppuration. Immediate incision, thorough disinfection, and drainage if necessary, relieve pain, shorten the duration and prevent extension of the inflammatory process.

In draining a suppurating wound, never cork it up by packing gauze in it. The smallest strip that will reach the bottom of the cavity, very loosely applied, is the best.

Constitutional treatment is all-important in all forms of diffuse surgical inflammations.

Recurrence of carbuncles and boils suggests an examination of the urine for diabetes.

See that patients have a good night's sleep the night before an operation.

Skin grafting will not succeed upon an unhealthy surface.

Watch patients with burns of the pharynx and larynx; be ready to operate at once. Severe dyspnea may occur with appalling suddenness. If the patient is getting cold and feeble, his ability to feel pain has greatly diminished. Waste no time in anesthesia in emergency tracheotomies.—*Maryland Medical Journal.*

